# Redescription of the enigmatic genus *Genuotermes* Emerson (Isoptera, Termitidae, Termitinae)

**DOI:** 10.3897/zookeys.340.6131

**Published:** 2013-10-04

**Authors:** Mauricio M. Rocha

**Affiliations:** 1 Museu de Zoologia da Universidade de São Paulo, Cx. Postal 42.494, 04218-970 São Paulo, SP, Brasil

**Keywords:** Taxonomy, morphology, worker gut anatomy, new distributional records, Neotropical region

## Abstract

The imago and soldier castes of the Neotropical Termitinae species *Genuotermes spinifer* Emerson are redescribed. The gut anatomy of the worker is described in detail for the first time, and morphological variations in the soldier are noted and illustrated. The known geographical distribution of *Genuotermes spinifer* is greatly expanded.

## Introduction

The subfamily Termitinae is represented in the Neotropical region by 18 genera and 96 species (Constantino 2013). Some Neotropical Termitinae genera were recently revised, and the remaining genera are very heterogeneous and poorly defined, much in need of redefinition of the specific limits.

The genus *Genuotermes* was first described by Emerson (1950) from a single soldier specimen collected under a log in the municipality of Corumbá, state of Mato Grosso do Sul, Brazil. Later, Mathews (1977) collected specimens of *Genuotermes spinifer* Emerson, the type-species of *Genuotermes*, inside *Cornitermes* nests in the Serra do Roncador, state of Mato Grosso, and described the imago and worker caste and redescribed the soldier caste, based on external morphological characters.

Re-examination of *Genuotermes* samples deposited in the MZUSP led to a revision of some of the diagnostic characters given in the earlier descriptions (Emerson 1950; Mathews 1977) and provides a set of morphological characters that distinguish *Genuotermes spinifer* from all other Neotropical Termitinae species. Notes and illustrations of morphological variations in the soldier caste are presented for the first time. Also, new distributional records are listed for *Genuotermes spinifer*, based on samples deposited in different Brazilian Isoptera collections.

## Material and methods

The material examined is deposited in the Isoptera collection of the Museu de Zoologia da Universidade de São Paulo (MZUSP). All examinated material are from Brazil and listed under the species redescription, arranged by state (in italics), and the corresponding lot number from the MZUSP (in parentheses). An asterisk after the lot number indicates the samples that contain imagoes. The GPS coordinates are indicated in decimal degrees, and only in the cases that have been registered by the own collectors. The records of samples deposited in the Museu Paraense Emílio Goeldi (MPEG), the Instituto Nacional de Pesquisas da Amazônia (INPA) and the Universidade de Brasília (UnB) were also included to compose a comprehensive species distribution map. The line drawings were made with a camera lucida, and the photographs were taken with a digital camera coupled to a stereomicroscope at different focal points and merged with software. The enteric valve and crop of a worker were mounted on a slide with glycerin and photographed under an optical microscope.

**Figures 1–2.**
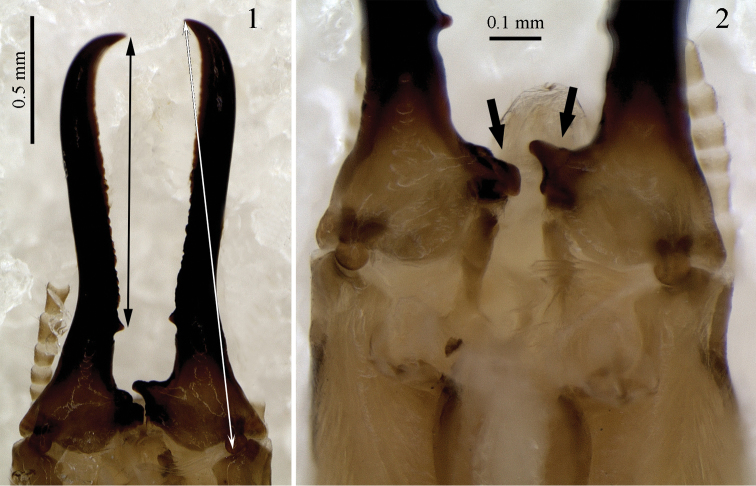
*Genuotermes spinifer*: Soldier mandibles in ventral view (mentum and maxillae removed): **1** Distance from the first marginal teeth to the apex (black arrow) and the cross length of mandible (white arrow) **2** Detail of the molar plate and prominence (arrows).

The terminology adopted for the worker digestive tube follows Noirot (2001). The morphometric characters used here and their correspondences with Roonwal’s system (Roonwal 1970) are indicated in parentheses, as follows: cross length of mandible, CLM (39); width of head capsule, WH (18); length of head capsule, LH (9); maximum width of pronotum, MWP (68); length of left hind tibia, LT (85). The “distance from the first marginal teeth to the apex” (DMA) is also included, as explained in Fig. 1 (black arrow), as also the cross length of mandible (white arrow). The maximum and minimum values follow the description (N=32 soldiers from all samples examined).

## Taxonomy

### Genuotermes

Emerson

http://species-id.net/wiki/Genuotermes

#### Type-species.

*Genuotermes spinifer* Emerson, by monotypy Emerson 1950: p. 5–6 (soldier, Fig. 2).

Mathews 1977: p. 121 (soldier redescription, no figures; imago description, figs 51, 63; worker description, plate 25).

#### Description.

**Figures 3–5.**
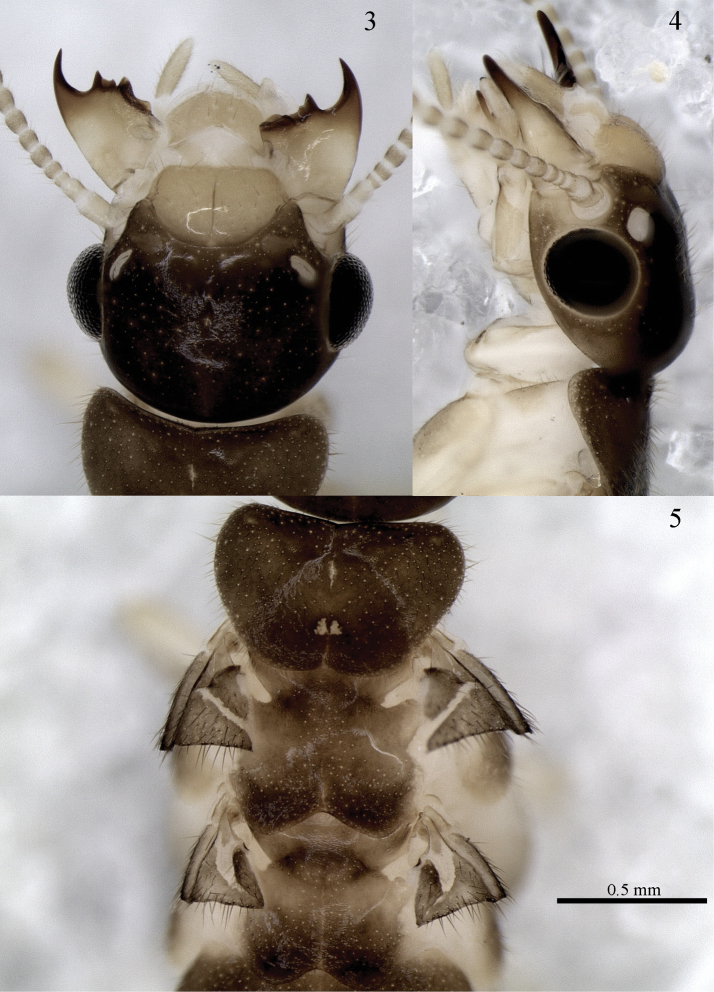
*Genuotermes spinifer* imago, female: **3** Dorsal view of head **4** Profile view of head **5** Pronotum and posterior margins in dorsal view.

*Imago*. Eyes semispherical, oval in profile, close to but not touching lower margin of head and ocellus (Fig. 4). Fontanelle indistinct, resembling a small slit on the dorsal surface of the head (Fig. 3). Pronotum subtrapezoidal, with anterolateral margins rounded. Posterior margin of mesonotum deeply notched and acute, margin of metanotum more shallow and rounded (Fig. 5). notched Formula for tibial spurs 3:2:2. Head and labrum covered with moderately dense layer of erect bristles, remainder of body covered with very dense layer of decumbent bristles.

**Figures 6–9.**
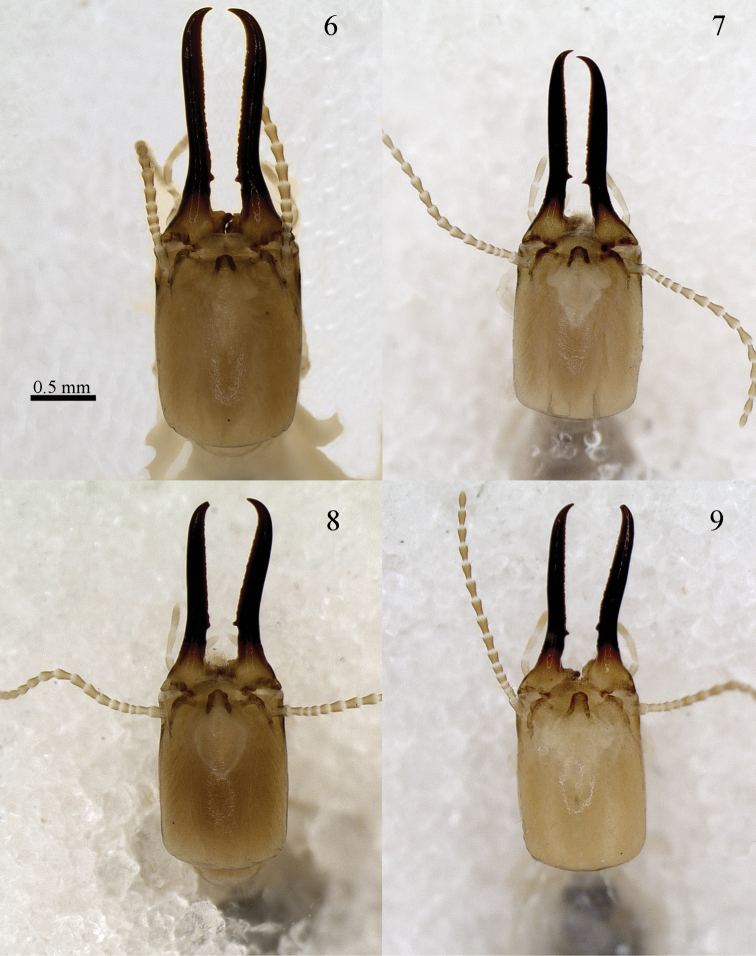
*Genuotermes spinifer*: Soldier morphological variations, dorsal view: **6** Fordlândia, PA (MZUSP-8383) **7** Chapada dos Guimarães, MT (MZUSP-6615) **8** Serra do Roncador, MT (MZUSP-7400) **9** UHE Santo Antônio (Módulo de Jirau), RO (MZUSP-16354).

**Figures 10–13.**
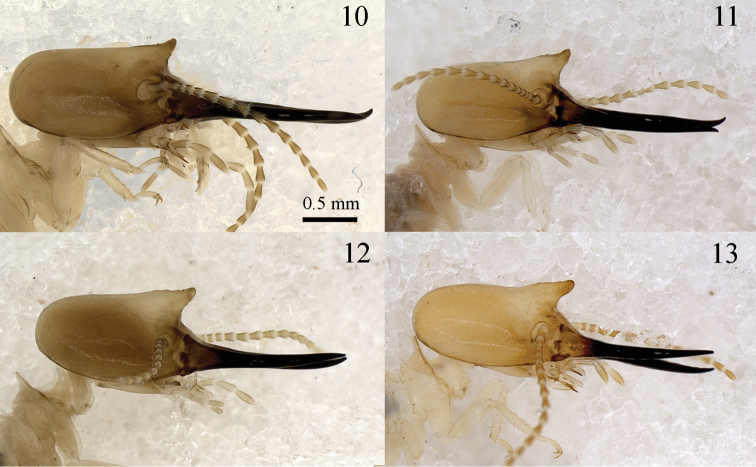
*Genuotermes spinifer*: Soldier morphological variations, profile: **10** Fordlândia, PA (MZUSP-8383) **11** Chapada dos Guimarães, MT (MZUSP-6615) **12** Serra do Roncador, MT (MZUSP-7400) **13** UHE Santo Antônio (Módulo de Jirau), RO (MZUSP-16354).

*Soldier*. Mandibles elongated, elbowed at proximal one-third, distal two-thirds with serrated blade on inner surface and salient tooth at base (Figs 1, 6–9). Molar plate and prominence visible at the base (Fig. 2, arrows). Head capsule subrectangular in dorsal view, with a characteristic frontal projection, best viewed in profile (Figs 10–13). Frontal gland aperture at tip of head projection, oriented anteriorly. Labrum subrectangular. Formula for tibial spurs 3:2:2. Head with very sparse bristles, denser on postclypeus and labrum surface, and around the frontal gland aperture. Pro-, meso- and metanotum with bristles only around all margins. Tergites, sternites and legs covered with bristles of variable length, denser than on remainder of body.

*Worker*. Head capsule rounded in dorsal view. Fontanelle inconspicuous. Antenna with 14–15 articles. Mandibles as in Fig. 19; apical tooth much more developed than marginal teeth, the latter with recognizable tips. Head capsule pilosity similar to imago; thorax and abdomen pilosity similar to soldier.

**Figures 14–19.**
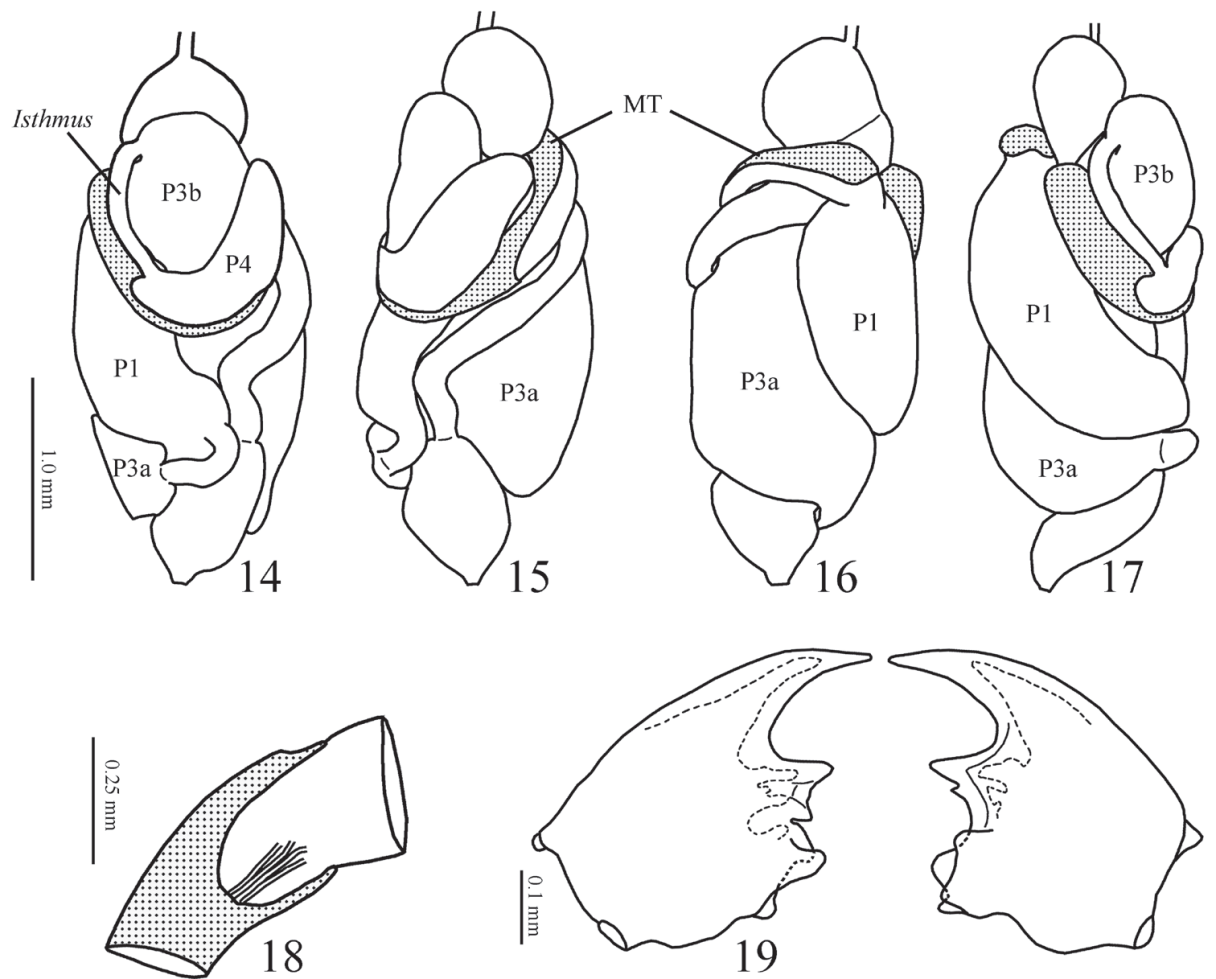
**14–17** *Genuotermes spinifer* worker gut *in situ*. **14** Dorsal **15** Right **16** Ventral **17** Left (MT = mesenteric tongue; P1 = first proctodeal segment (ileum); P3a and b = third proctodeal segment (paunch); P4a = first part of fourth proctodeal segment (colon) **18** Malpighian tubules insertion **19** Worker mandibles, dorsal view.

**Figures 20–21.**
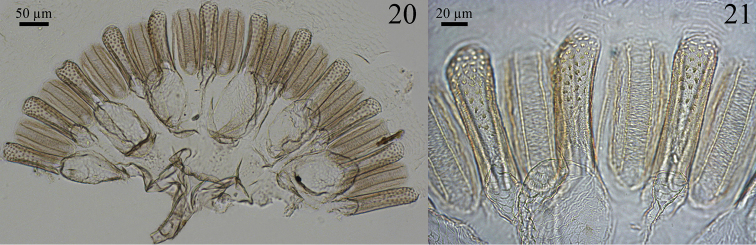
*Genuotermes spinifer* worker gizzard: **20** Columnar and pulvillar belts **21** Detail of ornamentation on the columns.

**Figures 22–23.**
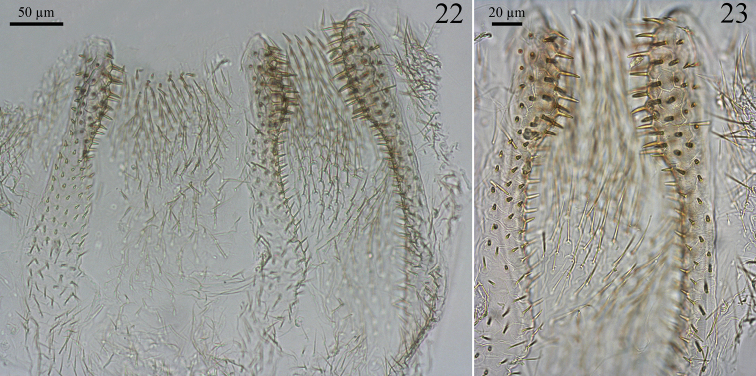
*Genuotermes spinifer* worker enteric valve: **22** Arrangement of the cushions **23** Detail of the cushions.

Digestive tube. Gizzard (Figs 20 and 21) having a columnar belt with 24 visible folds, six of the first order, six of second, and 12 of third, first-order folds with distinctive ornamentation of spines (Fig. 21), pulvilli of second order one-third as large as first-order pulvilli. Mesenteric tongue (MT) elongated, of uniform width, covering half length of mesenteric arch and facing anterodorsal region of body (Figs 15 and 16). Malpighian tubules inserted close to each other on inner surface of mesenteric arch (Fig. 18). P1 dilated, located on left side of body, with the final portion forming an elongated loop that reaches the dorsal region and down to insert on P3 (Fig. 14). Enteric valve (P2) composed by three equidistant longitudinal cushions (Fig. 22), slightly dilated at apex and covered with strong erect spines in a brushlike arrangement (Fig. 23), remaining surfaces with aciculiform and decumbent spines. P3a and P3b without a clear distinction, isthmus clearly recognizable, inserted subapically to beginning of P4 (Figs 14, 17). P4 short, situated in dorsal region of body.

#### Comparisons with other Neotropical genera of Termitinae

*Imago*. The imago of many species are still unknown and it is difficult to make a comparison for this caste, however some combination of characters may be useful for a diagnosis of this species. The mandibles are very characteristic, with the apical teeth well developed and very acute, the marginal teeth are all well recognizable (in some soil feeder species the M3 of both mandibles are not prominent) and the interval between the M3 and molar plate is narrow. Other unusual characteristics of this species are the thorax and abdomen covered only with decumbent bristles, without any erect setae or bristle.

*Soldiers*. *Genuotermes* soldiers have a combination of characters that make it easily distinguishable from other Neotropical Termitinae genera. *Genuotermes* and *Orthognathotermes* Holmgren 1910 (supposed by Emerson as the closest genera) have elongate mandibles, slightly elbowed outward and symmetrical, a unique characteristic among all Neotropical Isoptera; however, there are some clear differences between the two genera. *Genuotermes* has a projection on the anterior dorsal region of the head, which is absent in *Orthognathotermes*. The frontal gland aperture is recognizable at the apex of projection, whereas in *Orthognathotermes* the aperture is inconspicuous. In the remaining Termitinae genera with similar head structures (*Cavitermes* Emerson, 1925; *Cornicapritermes* Emerson, 1950; *Dihoplotermes* Araujo, 1961; *Divinotermes* Carrijo and Cancello, 2011; *Inquilinitermes* Mathews, 1977; *Spinitermes* Wasmann, 1897; *Termes* Linnaeus, 1758) the gland opening is situated on the base of their respective projections. In the *Genuotermes* soldier the molar plate and prominence are visible at the base of the mandibles (Fig. 2, arrows), in *Orthognathotermes* these structures are absent.

*Workers*. In the gut the differences among other Neotropical Termitinae genera are much more evident. In *Orthognathotermes* the P1 is tubular, with the same diameter as the mesenteron; the enteric valve (P2) insertion is situated on the right side of the body; and the armature is composed of cushions with projections to the P3 lumen (see Rocha and Cancello 2009: p. 20). In *Genuotermes* the P1 is more enlarged than the mesenteron, with the distal end narrowed, forming a short neck prior to the attachment to P3, and inserted on the left side of the body. This conformation is similar to *Microcerotermes* Silvestri, 1901, *Amitermes* Silvestri, 1901 (see Sands 1998: p. 640–668, 395), *Neocapritermes* Holmgren, 1912 (see Constantino 1998) and some Syntermitinae species (is particularly very similar to *Curvitermes*, see Carvalho and Constantino 1998: p. 650; and *Silvestritermes*, see Rocha et al. 2012: p. 811–812). The enteric valve armature is very similar to *Neocapritermes*, with columnar cushions arranged triradially (Fig. 22), but, some Syntermitinae species apparently share this triradial digitiform pattern (*Paracurvitermes*, see Constantino and Carvalho 2011 p. 285; *Cyrilliotermes*, Constantino and Carvalho 2012 p. 38 and *Embiratermes festivellus*, see Rocha et al. 2012: p. 801), although not so similar.

### Genuotermes spinifer

Emerson

http://species-id.net/wiki/Genuotermes_spinifer

Figs 1 –24

#### Holotype.

One soldier deposited at the American Museum of Natural History (AMNH), from Brazil, Mato Grosso do Sul state, Serra do Urucum (Municipality of Corumbá), 14.viii.1926, K.P. Schmidt coll. (not examined).

#### Imago.

As for the genus (*vide supra*). *Measurements*. See Mathews (1977: 124).

#### Soldier.

As for the genus (*vide supra*). *Measurements* (mm): DMA: 1.05–1.40; CLM: 1.65–1.95; WH: 1.05–1.25; LH: 1.50–1.75; MWP: 0.68–0.93; LT: 0.85–1.00.

#### Worker.

As for the genus (*vide supra*).

#### Biology and habits.

Information about the life habits of *Genuotermes* is sparse and comes only from field notes of collectors. This species apparently lives in diffuse galleries in the soil, and is sometimes found in the nest of other termites (*Labiotermes leptothrix*, *Cornitermes cumulans* and *Cornitermes silvestrii*). They probably feed on humus in subterranean galleries. The morphology of the worker mandibles (humivorous type) and the finding of sand particles in the gut (personal observation) concord with this hypothesis.

#### Geographical distribution.

The records of *Genuotermes spinifer* presented here represent a significant extension of the previously known distribution. The species’ occurrence now includes different vegetation zones, the Brazilian Cerrado and the Amazon Forest.

**Figure 24.**
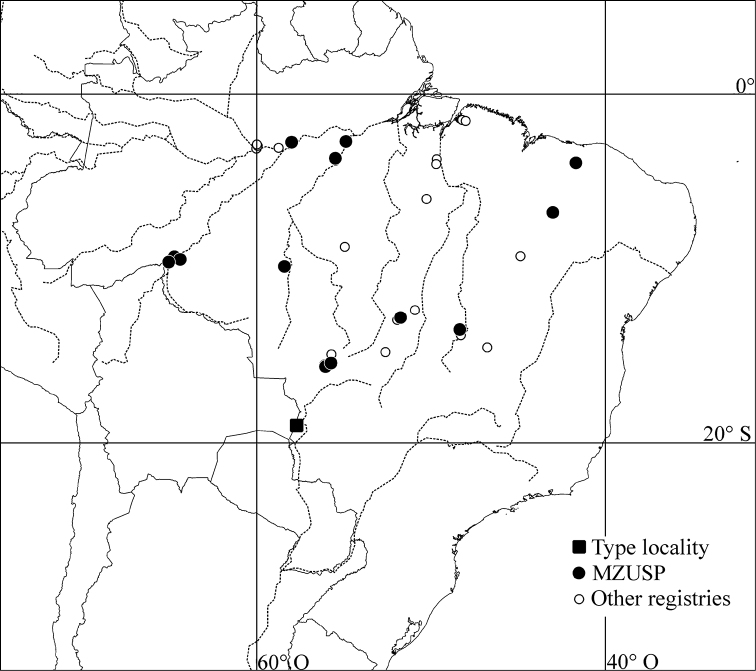
Geographic records of *Genuotermes spinifer* from type-locality (black square), material examined from MZUSP (black circle), and samples deposited at INPA, MPEG and UnB.

#### Material examined.

BRAZIL. *Amazonas*: Itacoatiara, 04.xi.1977, A. Bandeira (7479); 23.v.1977 (8353). *Goiás*: Cana Brava (Faz. Itaúna), 07.v.1975, K. Kitayama (7310). *Mato Grosso*: Cuiabá (14 Km NW), 15.ii.1976, R.L. Araujo (6526); Cuiabá (Vale do Rio Mutuca), 06-10.viii.2009, S.P. Rosa (12643); Chapada dos Guimarães, 10.ii.1976, R.L. Araujo (6633, 6615); Cotriguaçu (-9.857; -58.413), 29.xi.2011, R.C. Paula (16352); Coxipó, 14.ii.1976, R.L. Araujo (6703); 18.ii.1976 (7217); 17.ii.1976 (6857); Serra do Roncador, ix–x.1968, A.G.A. Mathews (7400, 7399*). *Pará*: Belterra, 31.i.1949, C.R. Gonlçalvez (4306); Fordlândia, ii.1957, A.M. Almeida (8383). *Piauí*: Floriano, 5–12.xi.1991, E.M. Cancello and M.T. Ponte (10188); Sete Cidades, 14.xii.1976, R.L. Araujo (7194). *Rondônia*: Porto Velho (Hydroelectric Reservoir of Jirau, Mutum-Paraná, -9.607; -65.050), 26.ii–13.iii.2010, T. Carrijo and R. Santos (13009); Porto Velho (UHE Santo Antônio, Jaci-Paraná, -9.4559; -64.388), 08.iv.2011, R. Santos and C. Mandai (16355); 09.ix.2011, R. Santos and J. Cabral (16253); 06.iii.2012, T. Carrijo and J. Cabral (16356); Porto Velho (UHE Santo Antônio, Jirau, -9.312; -64.726), 18.ix.2010, T. Carrijo and R. Santos (16354*).

## Supplementary Material

XML Treatment for Genuotermes

XML Treatment for Genuotermes spinifer
